# eIF2α signaling regulates ischemic osteonecrosis through endoplasmic reticulum stress

**DOI:** 10.1038/s41598-017-05488-6

**Published:** 2017-07-11

**Authors:** Daquan Liu, Yunlong Zhang, Xinle Li, Jie Li, Shuang Yang, Xiaoxue Xing, Guanwei Fan, Hiroki Yokota, Ping Zhang

**Affiliations:** 1 Department of Anatomy and Histology, School of Basic Medical Sciences, Tianjin Medical University, Tianjin, 300070 China; 2 Department of Pharmacology, Institute of Acute Abdominal Diseases, Tianjin Nankai Hospital, Tianjin, 300100 China; 3 TEDA International Cardiovascular Hospital, Chinese Academy of Medical Sciences & Peking Union Medical College, Tianjin, 300457 China; 4 School of Stomatology, Tianjin Medical University, Tianjin, 300070 China; 5 Key Laboratory of Hormones and Development (Ministry of Health), Tianjin Key Laboratory of Metabolic Diseases, Tianjin Medical University, Tianjin, 300070 China; 6 State Key Laboratory of Modern Chinese Medicine, Tianjin University of Traditional Chinese Medicine, Tianjin, 300193 China; 7 Department of Biomedical Engineering, Indiana University-Purdue University Indianapolis, Indianapolis, IN 46202 USA

## Abstract

Osteonecrosis of the femoral head (ONFH) primarily results from ischemia/hypoxia to the femoral head, and one of the cellular manifestations is the endoplasmic reticulum (ER) stress. To understand possible linkage of ischemic osteonecrosis to the ER stress, a surgery-induced animal model was employed and salubrinal was administered to evaluate the role of ER stress. Salubrinal is a synthetic chemical that inhibits de-phosphorylation of eIF2α, and it can suppress cell death from the ER stress at a proper dose. The results indicated that the ER stress was associated with ONFH and salubrinal significantly improved ONFH-induced symptoms such as osteonecrosis, bone loss, reduction in vessel perfusion, and excessive osteoclastogenesis in the femoral head. Salubrinal also protected osteoblast development by upregulating the levels of ATF4, ALP and RUNX2, and it stimulated angiogenesis of endothelial cells through elevating ATF4 and VEGF. Collectively, the results support the notion that the ER stress is an important pathological outcome in the surgery-induced ONFH model, and salubrinal improves ONFH symptoms by enhancing angiogenesis and bone healing via suppressing the ER stress.

## Introduction

The endoplasmic reticulum (ER) is a cellular organelle for modifying, folding, and transporting various proteins^1^. When ER homeostasis is disturbed, for example by ischemia/hypoxia, the unfolded protein response (UPR) takes place followed by induction of the stress to the ER^1, 2^. When cells experience the prolonged and unmitigated ER stress, they undergo apoptosis^1, 3^. Although the ER stress has been reported to be linked to various diseases such as diabetes^4^, neurodegenerative diseases^5^, osteoporosis^6^, and osteogenesis imperfecta^7^, the role of the ER stress in the pathogenesis of ischemic bone diseases remains unclear.

Ischemia/hypoxia is known to induce the ER stress^8^ and increases mortality, for instance, in the mouse model of acute myocardial infarction^9^. However, little is known about whether ischemic osteonecrosis of the femoral head (ONFH) triggers the ER stress, and whether any pharmacological approach for suppressing the ER stress may mitigate symptoms. ONFH, a serious orthopedic problem in the hip joint, results from an impaired blood supply to the femoral head^10, 11^. Its etiologic risk factors include trauma^12, 13^, excessive alcohol intake^14^, corticosteroid use^15–17^, and Legg-Calve-Perthes disease^18, 19^. A lack of proper blood circulation results in necrosis, followed by a collapse of the femoral head^20^. In the United States, approximately 50,000 hip replacements are annually performed for patients with ONFH^21^. Surgical therapies, including autologous bone marrow transplantation, core decompression osteotomies, vascularized or non-vascularised bone grafting, and total hip replacement, are primary treatments for the end-stage ONFH^22^. Non-surgical therapies include drugs for anticoagulation and anti-osteoporosis, a reduction in weight bearing, hyperbaric oxygen therapy, and electromagnetic and shockwave stimulation^23, 24^. Because of limited efficacy and potential side effects, however, few non-surgical therapies are satisfactory. In this study, we examined whether the ER stress is triggered in the ischemic ONFH, and evaluated whether alleviating the ER stress may provide a novel pharmacological approach in the treatment for ONFH.

Eukaryotic translation initiation factor 2 subunit alpha (eIF2α)^25, 26^ is an important member of protein synthesizing machinery in the regulatory axis in the ER stress that involves protein kinase RNA-like ER kinase (PERK) and activating transcription factor 4 (ATF4). Following the accumulation of misfolded proteins, PERK is activated after dissociation of glucose-regulated protein 78 (GRP78) and attenuates protein synthesis by phosphorylating eIF2α. ATF4 induces anti-stress responses by promoting transcription of specific genes such as ER chaperones (such as GRP78) and leading to, for instance, autophagy. Besides the PERK/ATF4 axis, other regulators include growth arrest and DNA damage inducible protein (GADD34) and CCAAT/enhancer binding protein homologous protein (CHOP). Activation of GADD34 regulates a recover process from the inhibition of protein synthesis. In response to the prolonged and unmitigated ER stress, CHOP may induce apoptosis by stimulating pro-apoptotic factors (e.g., death receptor 5, DR5) and blocking anti-apoptotic B cell CLL/lymphoma 2 (BCL-2)^26^.

Salubrinal (480 Da, C_21_H_17_Cl_3_N_4_OS) is a selective inhibitor of protein phosphatase I (PP1) complex, which de-phosphorylates eIF2α^25, 26^. Salubrinal is known to regulate bone metabolism by elevating phosphorylated eIF2α (p-eIF2α) and activating transcription factors such as ATF4^27^. It is reported that salubrinal stimulates bone healing, and serves as a potential drug candidate for treatment of osteoarthritis and osteoporosis by reducing joint inflammation and bone loss^27–29^. We have recently demonstrated that the ER stress plays a key role in the pathogenesis of disuse osteoporosis, and salubrinal attenuates unloading-induced bone loss by altering the proliferation and differentiation of osteoblasts and osteoclasts^30^. In the present study, we investigated whether the ER stress is triggered in the ischemic ONFH, and whether salubrinal is effective in the treatment of ONFH. We employed a rat model of surgically induced ONFH, and evaluated angiogenesis and bone remodelling by examining fates of endothelial cells, osteoclasts, and osteoblasts.

## Results

### Salubrinal has no significant effects on bone metabolism of normal animals

The body weight of animals in the sham control and salubrinal-treated sham control groups were not significant difference (*P* > 0.05, Supplemental Fig. 1a). For pDEXA scanning, BMD and BMC of the femurs in these two groups were not significant difference (*P* > 0.05, Supplemental Fig. 1a,b). The images of H&E staining indicated that the general structure and B.Ar/T.Ar of the femoral heads were not significant difference in these groups (*P* > 0.05, Supplemental Fig. 1d,e). The result indicated that salubrinal had no significant effects on bone metabolism of normal animals. We thus did not include salubrinal-treated sham control groups in the subsequent experiments.

### Salubrinal suppresses bone loss in ONFH

**Figure 1:**
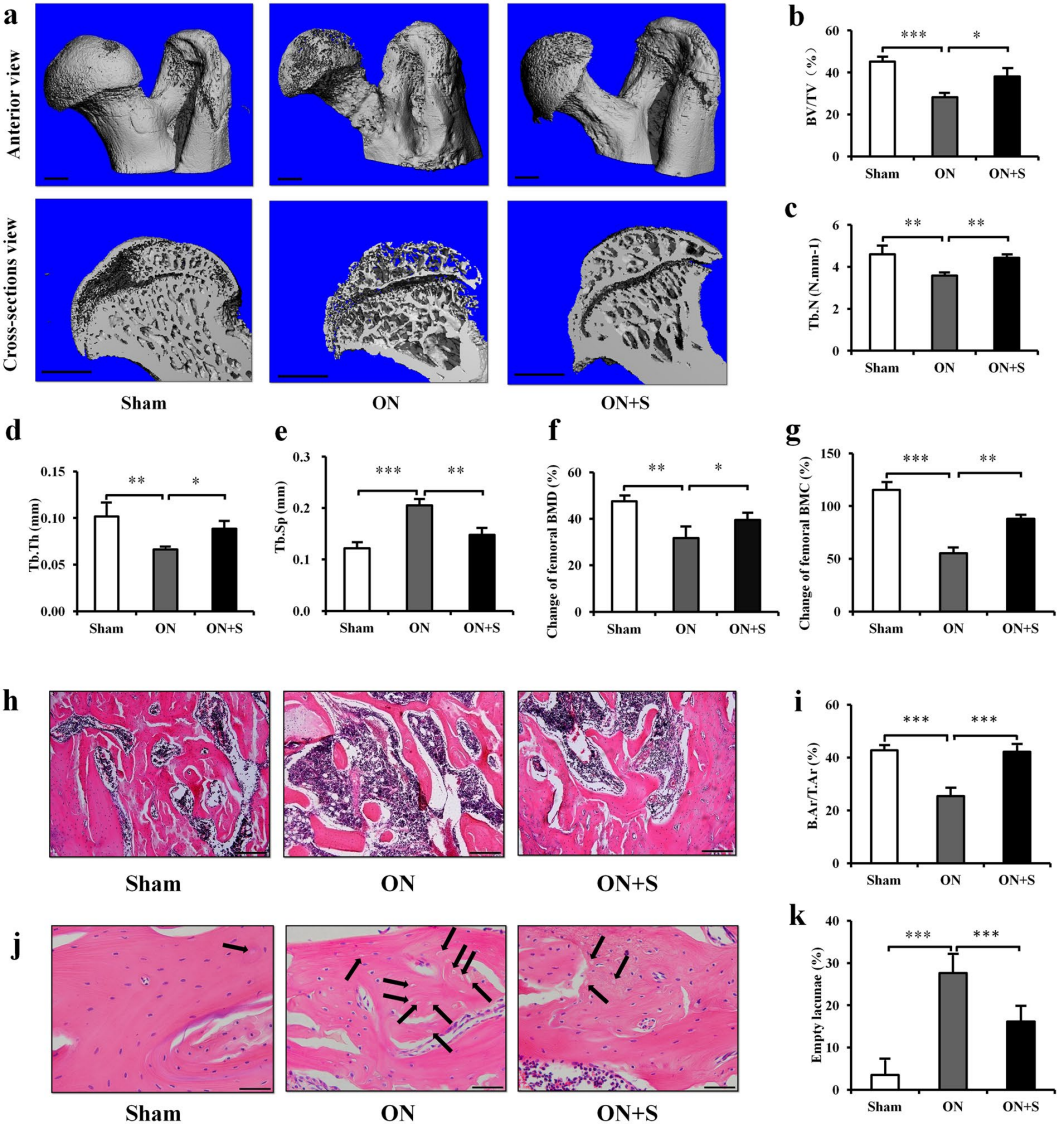
Salubrinal suppresses bone loss in ONFH. (**a**) Representative μ-CT reconstructed image of the femoral head (Bar = 1 mm). (**b**–**e**) Architectural properties in ONFH were measured by μ-CT. (**b**) Fractional bone volume (BV/TV). (**c**) Trabecular number (Tb.N). (**d**) Trabecular thickness (Tb.Th). (**e**) Trabecular spacing (Tb.Sp). (**f**) Bone mineral density (BMD). (**g**) Bone mineral content (BMC). (**h**–**j**) Representative histological images of the femoral head by H&E staining (**h**) 100×, Bar = 200 μm, and (**j**) 400×, Bar = 50 μm. Empty lacunae were indicated by the arrows. (**i**) Ratio of the trabecular bone area to the tissue area (B.Ar/T.Ar). (**k**) Percentage of empty lacunae. The asterisks (*, **, and ***) represent *P* < 0.05, *P* < 0.01, and *P* < 0.001, respectively (n = 12) (Sham: sham control group, ON: osteonecrosis group, ON + S: salubrinal treated osteonecrosis group).

In the sham control group, the femoral head and neck surface was not detectably altered. However, the femoral head surface in the ON group exhibited “moth eaten” appearance and the femoral neck was thinned. Compared to the ON group, the salubrinal-treated group suppressed osteonecrosis-driven bone loss in the head and neck (Fig. 1a). In the ON group, the values of BV/TV, Tb.N, Tb.Th, and BMD/BMC were decreased, and the value of Tb.Sp was increased (all *P* < 0.01). Salubrinal significantly increased the values of BV/TV, Tb.N, and Tb.Th, as well as the change of femoral BMD/BMC. It also decreased the value of Tb.Sp (all *P* < 0.05; Fig. 1b–g). Regarding the results of H&E staining in the femoral head (Fig. 1h,j), the ON group exhibited the lowest B.Ar/T.Ar with the highest percentage of empty lacunae (both *P* < 0.001). The salubrinal-treated group significantly improved those values (both *P* < 0.001; Fig. 1i,k).

### Salubrinal inhibits osteoclastogenesis in ONFH

**Figure 2:**
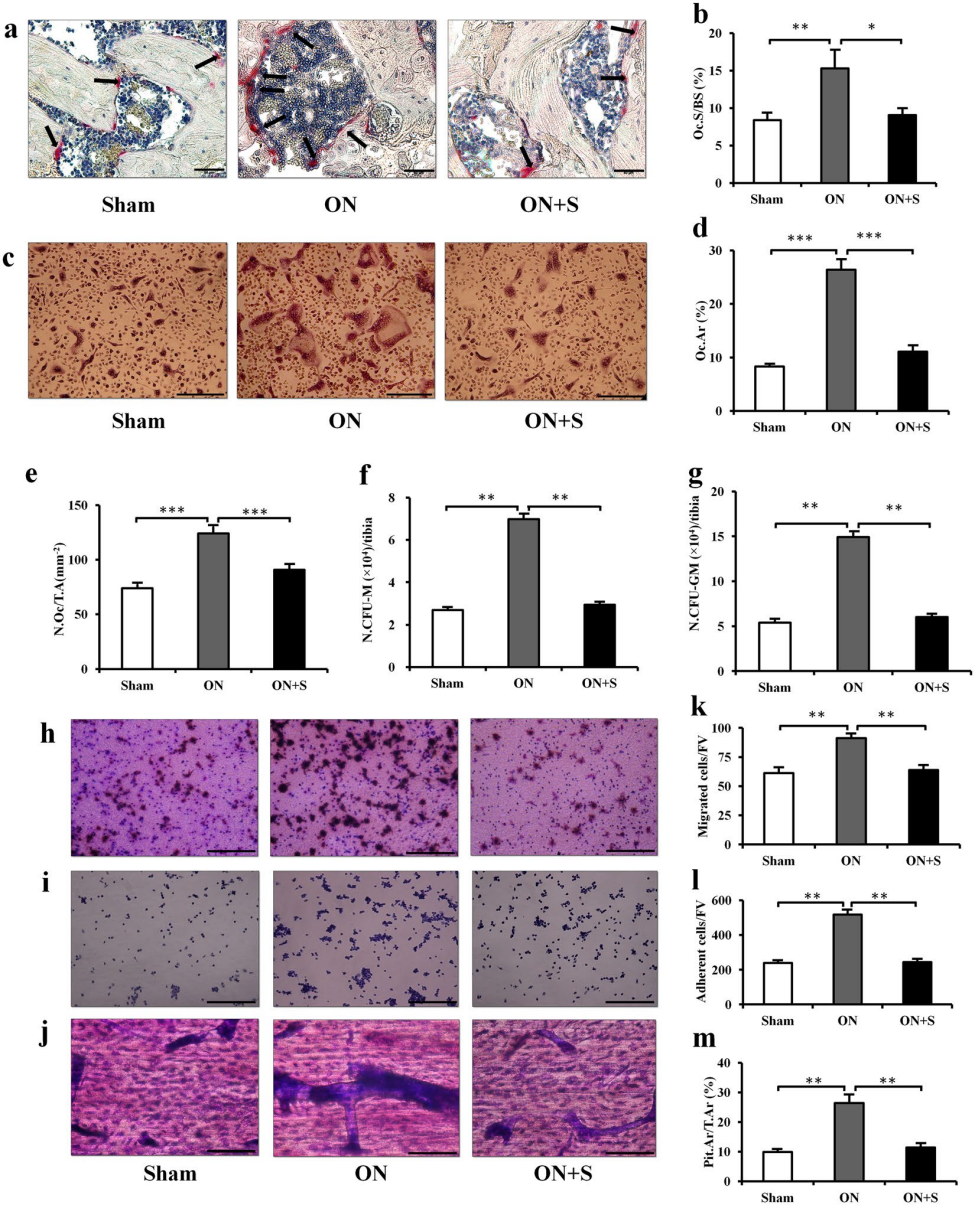
Salubrinal inhibits osteoclastogenesis in ONFH. (**a**) Representative histological images of the femoral head by TRAP staining. TRAP-positive cells, in red color, indicated by the arrows (400×, Bar = 50 μm). (**b**) Osteoclast surface (%). (**c**) Representative images of mature osteoclasts formation *in vitro* by TRAP staining (200×, Bar = 100 μm). (**d**) Percentage of the matured osteoclasts area. (**e**) Matured osteoclasts number. (**f**) CFU-M. (**g**) CFU-GM. (**h**) Representative images of pre-osteoclasts migration by crystal violet staining (200×, Bar = 100 μm). (**i**) Number of migratory pre-osteoclasts. (**j**) Representative images of pre-osteoclasts adhesion with crystal violet staining (200×, Bar = 100 μm). (**k**) Numbers of adherent pre-osteoclasts. (**l**) Representative images of bone resorption capacity of osteoclasts with crystal violet staining (400×, Bar = 50 μm). (**m**) Percentages of the pit area to total area in each field view from three groups. The asterisks (*, **, and ***) represent *P* < 0.05, *P* < 0.01, and *P* < 0.001, respectively (n = 12).

To investigate whether salubrinal affected the bone resorption of osteoclasts in ONFH *in vivo*, we conducted TRAP staining (Fig. 2a). The osteoclast surface (%) was increased in the ON group (*P* < 0.01), while this increase was suppressed in the salubrinal-treated group (*P* < 0.05; Fig. 2b). Regarding TRAP-positive multinuclear cells (more than 3 nuclei) identified as matured osteoclasts in osteoclast formation *in vitro* assay (Fig. 2c), the ON group significantly increased the osteoclast area (Fig. 2d) and the osteoclast number (Fig. 2e) (both *P* < 0.001), but the salubrinal-treated group significantly suppressed their increases (both *P* < 0.001). To determine whether salubrinal affected the proliferation and population of osteoclast progenitors, we counted CFU-M/CFU-GM. The numbers of CFU-M/CFU-GM were increased in the ON group (both *P* < 0.01), while salubrinal significantly reduced them (both *P* < 0.01; Fig. 2f,g).

To evaluate whether salubrinal affected the function of osteoclast lineage in ONFH, we performed migration, adhesion, and pit formation assays. In the ON group, migration (Fig. 2h) and adhesion (Fig. 2i) of pre-osteoclasts were stimulated, and an increased capacity for bone resorption was observed in the pit formation assay (all *P* < 0.01; Fig. 2j). Those activities were significantly reduced in the salubrinal-treated group (all *P* < 0.01; Fig. 2k–m).

### Salubrinal promotes osteoblast differentiation in ONFH

**Figure 3:**
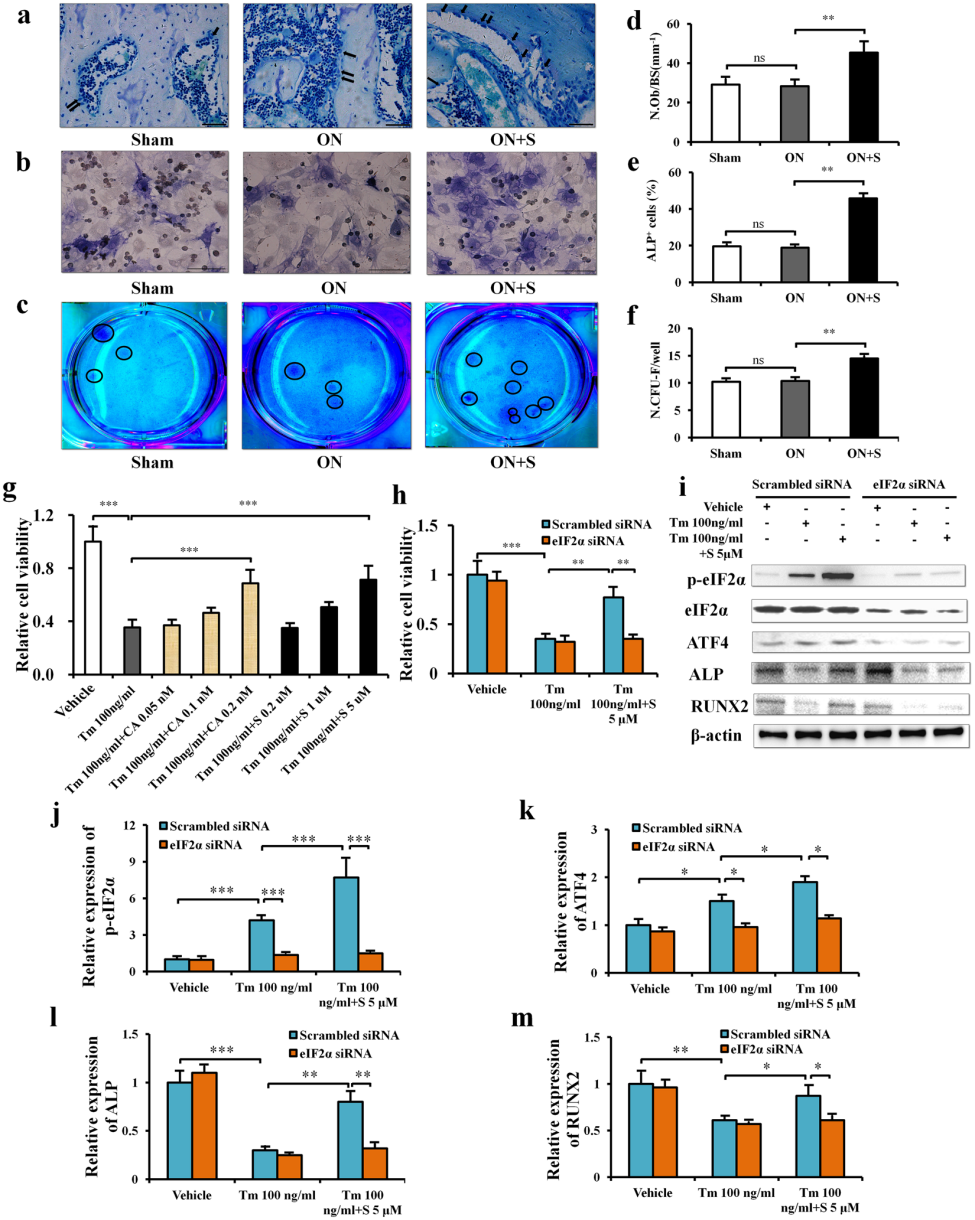
Salubrinal promotes osteoblast differentiation in ONFH. (**a**) Representative histological images of the femoral head by MacNeal’s staining. Osteoblasts, located on trabecular surface, were indicated by the arrows. (400×, Bar = 50 μm). (**b**) Quantification of the osteoblast number normalized to the total trabecular surface. (**c**) Representative images of osteoblast differentiation *in vitro* by ALP staining (400×, Bar = 50 μm). (**d**) ALP^+^ cells (%). (**e**) Colonies of fibroblasts stained using a HEMA-3 quick staining kit. (**f**) Numbers of colonies with more than 50 cells (the colonies were indicated by the circles). (**g**,**h**) Statistical analysis for cell viability of MC3T3-E1 cells. (**i**) Representative images of Western blotting for salubrinal’s protection of osteogenesis against the ER stress. The levels of p-eIF2α (**j**), ATF4 (**k**), ALP (**l**), and RUNX2 (**m**) are shown. The experiment was conducted in triplicate. The asterisks (*, ** and ***) represent *P* < 0.05, *P* < 0.01, and *P* < 0.001, respectively, and “ns” represents *P* > 0.05 (Tm: Tunicamycin, S: Salubrinal, and CA: Calyculin A).

To evaluate the differentiation of osteoblasts and fibroblasts from mesenchymal stem cells, osteoblasts were identified using MacNeal’s staining *in vivo* (Fig. 3a) or ALP staining *in vitro* (Fig. 3b), and fibroblasts were stained using a HEMA-3 quick staining kit. The number of osteoblasts (Fig. 3a,b) and the number of CFU-F (Fig. 3c) were not significantly different in the sham control and ON groups (all *P* > 0.05). However, the numbers were significantly increased in the salubrinal-treated group (all *P* < 0.01; Fig. 3d–f).

### Salubrinal promotes osteogenesis by increasing p-eIF2α *in vitro*

To evaluate whether salubrinal improved osteogenesis against the ER stress, cell viability and the expression of osteoblast markers were determined. Tunicamycin was used to activate ER stress signaling, and Calyculin A (CA, a specific inhibitor of PP1) was employed as a positive control to salubrinal^25^. We first evaluated the effects of tunicamycin, salubrinal, and CA to MC3T3-E1 cells. The result indicated that the non-toxic dose of salubrinal was 0.2 to 5 μM, while that for CA was 0.05 to 0.2 nM. We thereafter employed those concentrations, and evaluated their effects in the presence of tunicamycin. Tunicamycin inhibited viability of MC3T3-E1cells in a dose-dependent manner at 0.05, 0.1, 0.25, 0.5, 1, and 2.5 μg/ml. Interestingly, administration of 5 μM salubrinal or 0.2 nM CA partially restored cell viability in the presence of 100 ng/ml tunicamycin (both *P* < 0.001; Fig. 3g). However, salubrinal or CA was unable to rescue MC3T3-E1 cells at higher concentrations of tunicamycin (above 250 ng/ml, all *P* > 0.05; Supplemental Fig. 2a).

To investigate the role of eIF2α in the effect of salubrinal on osteogenesis, the cell viability and the expression of osteoblast markers were determined in MC3T3-E1 cells using eIF2α RNA interference. Transfection with eIF2α siRNA blocked the effect of salubrinal on cells viability under ER stress (*P* < 0.01; Fig. 3h). Furthermore, the protein levels of p-eIF2α, ATF4, ALP and RUNX2 in MC3T3-E1 cells were examined at 48 h after administration of 100 ng/ml tunicamycin with 5 μM salubrinal (Fig. 3i). Tunicamycin significantly inhibited the levels of ALP and RUNX2, while salubrinal increased the levels of p-eIF2α, ATF4, ALP, and RUNX2 (all *P* < 0.01). Compared to scrambled siRNA, eIF2α siRNA blocked the effect of salubrinal on the expression of p-eIF2α, ATF4, ALP, and RUNX2 under ER stress (all *P* < 0.05; Fig. 3j–m).

### Salubrinal elevates vessel density in ONFH

**Figure 4:**
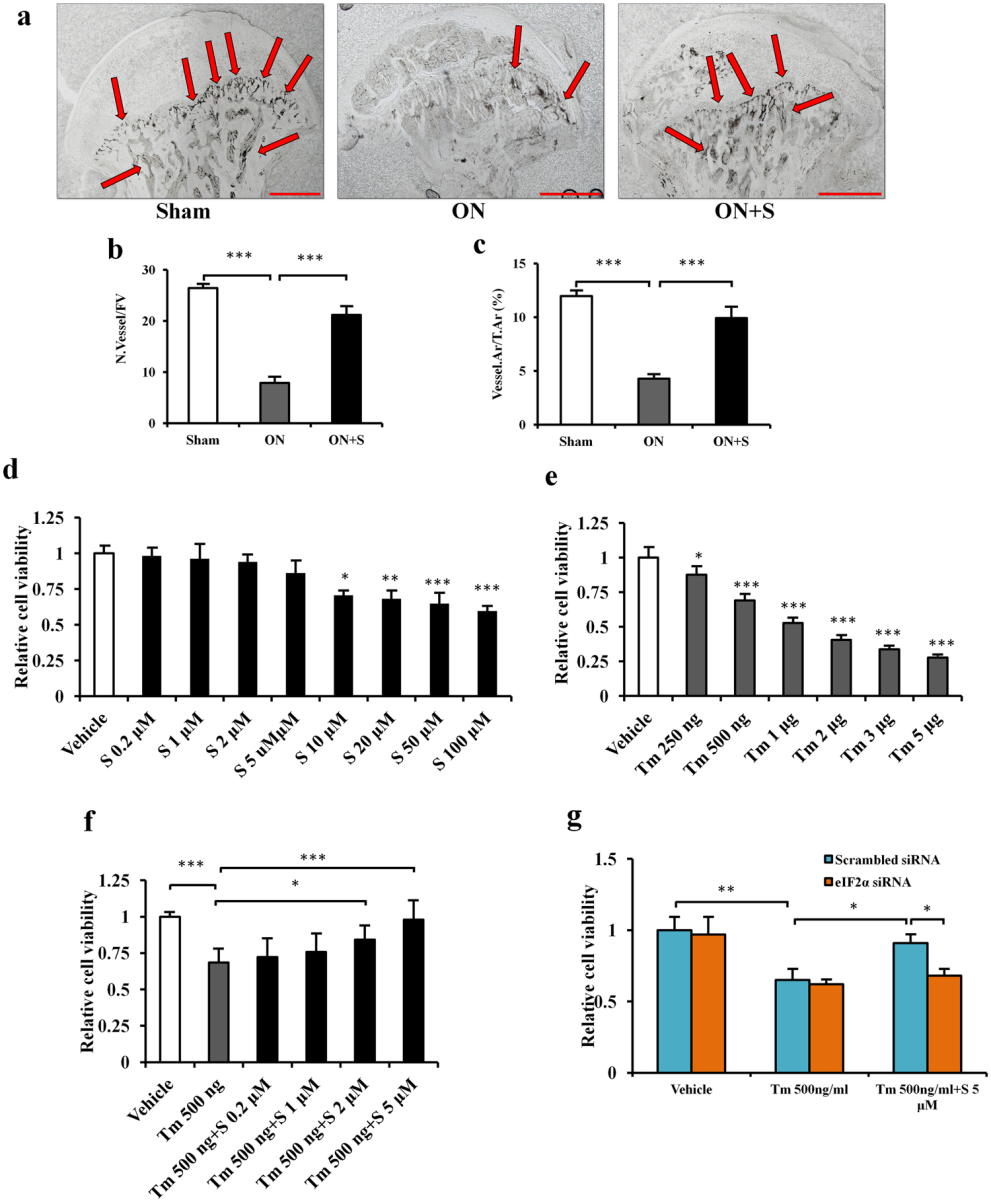
Salubrinal elevates blood perfusion in ONFH and protects viability of endothelial cells against ER stress. (**a**) Representative histological images of the femoral head by ink perfusion angiography. The vessels were indicated by the arrows (40×; Bar = 1 mm). The quantification of the vessel number (**b**) and vessel area percentage (**c**) in each field of view (200×) was conducted. (**d**–**g**) MTT assay for cell viability of HUVECs. The histogram showed relative cell viability from three independent experiments. The asterisks (*, ** and ***) represent *P* < 0.05, *P* < 0.01, and *P* < 0.001, respectively (S: Salubrinal, and Tm: Tunicamycin).

To investigate whether salubrinal improves blood perfusion in the ischemic femoral head, we determined the vessels in the femoral head using ink perfusion angiography (Fig. 4a). The ON group decreased the vessel numbers and vessel area (both *P* < 0.001), while the salubrinal-treated group improved them (both *P* < 0.001; Fig. 4b,c).

### Salubrinal protects endothelial cells by elevating p-eIF2α *in vitro*

In response to salubrinal, cell viability of human umbilical vein endothelial cells (HUVECs) was lowered at the concentration above 10 μM (all *P* < 0.05), but no detectable change in cell viability was observed at 0.2 to 5 μM (Fig. 4d). Thus, we employed 0.2 to 5 μM salubrinal to HUVECs and evaluated salubrinal’s effect in the presence of an ER stress inducer, tunicamycin. Tunicamycin inhibited cell viability of HUVECs in a dose-dependent manner (from 250 ng/ml to 5 μg/ml, all *P* < 0.05; Fig. 4e). Administration of 5 μM salubrinal restored cell viability in the presence of 500 ng/ml tunicamycin (*P* < 0.05; Fig. 4f). However, 5 μM salubrinal was unable to rescue the viability of HUVECs that were treated with higher concentrations of tunicamycin (more than 1 μg/ml, all *P* > 0.05, Supplemental Fig. 3a–c). The results indicated that 500 ng/ml tunicamycin induces significant responses in HUVECs. The dose of 500 ng/ml tunicamycin was thus used to induce ER stress in HUVECs. Furthermore, compared to scrambled siRNA, eIF2α siRNA transfection blocked the effect of salubrinal for restoring cell viability of HUVECs in the presence of 500 ng/ml tunicamycin (all *P* < 0.05; Fig. 4g).

**Figure 5:**
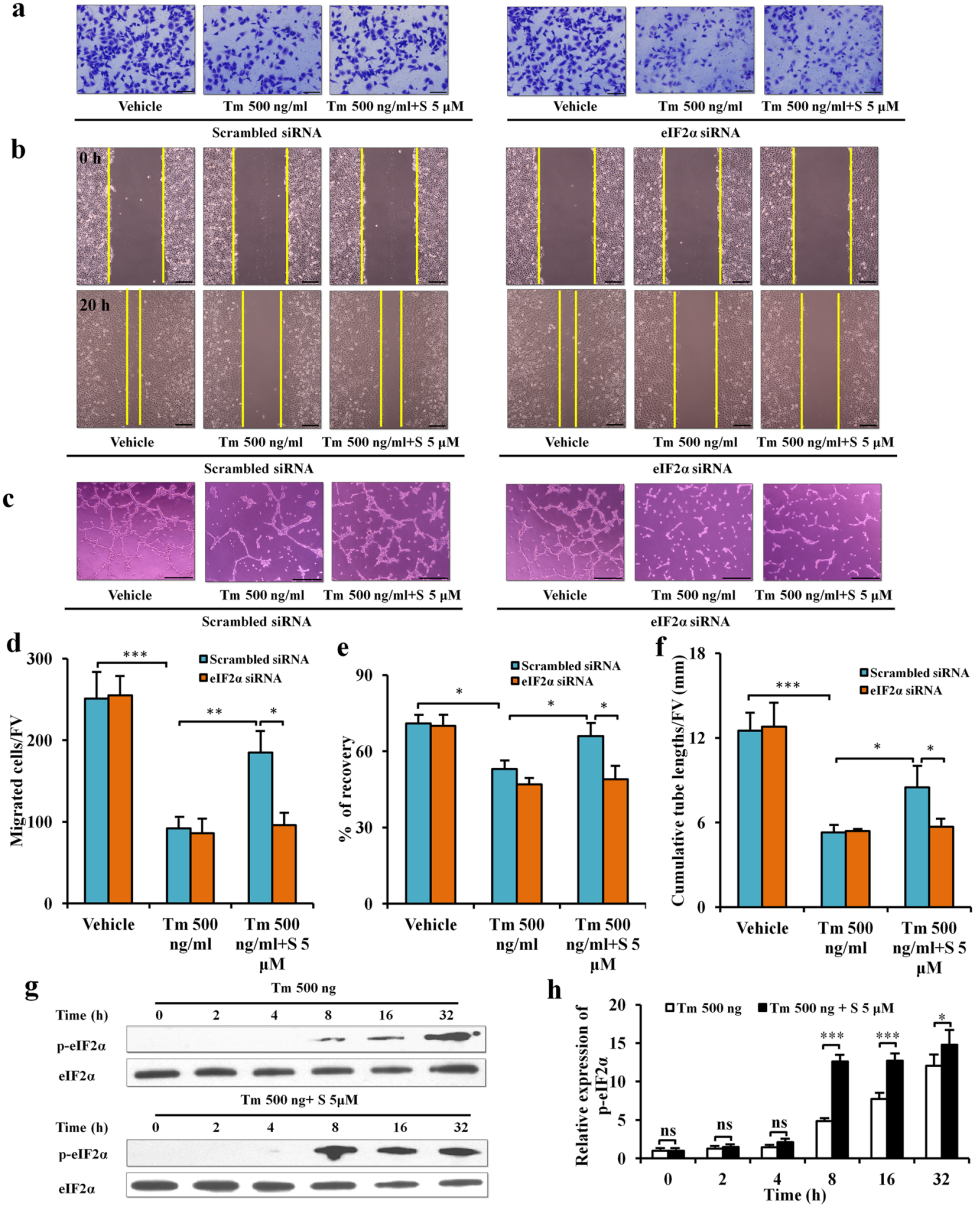
Salubrinal improves migration and angiogenesis of endothelial cells against ER stress by elevating p-eIF2α *in vitro*. (**a**) Representative images of HUVECs migration by crystal violet staining (200×, Bar = 100 μm). (**b**) Representative images of HUVECs wound-healing (100×, Bar = 200 μm). (**c**) Representative images of HUVECs tube formation (100×, Bar = 200 μm). (**d**) Numbers of migratory HUVECs. (**e**) Percentages of HUVECs wound recovery. (**f**) Cumulative tube lengths. The experiments were repeated on three independent times. (**g**) Level of p-eIF2α in HUVECs in the presence of tunicamycin and salubrinal at 6 time points (0, 2, 4, 8, 16, and 32 h). (**h**) Ratio of p-eIF2α to total eIF2α. The asterisks (*, **, and ***) represent *P* < 0.05, *P* < 0.01, and *P* < 0.001, respectively.

To investigate whether salubrinal affected migration and angiogenesis of HUVECs in the presence of tunicamycin, we performed assays for migration, wound healing and tube formation *in vitro*. In the migration (Fig. 5a) and wound healing (Fig. 5b) assays, tunicamycin inhibited the migration and wound recovery of HUVECs (both *P* < 0.05). Salubrinal rescued migration and wound recovery of HUVECs in the presence of tunicamycin (both *P* < 0.05). However, compared to scrambled siRNA, eIF2α siRNA transfection blocked the effects of salubrinal on migration and wound recovery of HUVECs under ER stress (all *P* < 0.05; Fig. 5d,e). In the tube formation assay (Fig. 5c), tunicamycin decreased cumulative tube lengths (*P* < 0.001), while salubrinal suppressed tunicamycin’s effect (*P* < 0.05). In accord with the migration, eIF2α siRNA blocked the effect of salubrinal on tube formation of HUVECs in the presence of tunicamycin (*P* < 0.05; Fig. 5f).

To evaluate whether salubrinal affected the p-eIF2α in HUVECs against tunicamycin, we determined the level of p-eIF2α in HUVECs at 0, 2, 4, 8, 16, and 32 h after administration of tunicamycin and salubrinal. Tunicamycin elevated the level of p-eIF2α at 8 h and peaked it at 32 h. Salubrinal increased the level of p-eIF2α at 4 h, and this elevation was kept at 8, 16, and 32 h. Compared to non-salubrinal treatment, salubrinal administration increased the level of p-eIF2α in the presence of tunicamycin at 8, 16, and 32 h (all *P* < 0.05; Fig. 5g,h).

### Salubrinal increases p-eIF2α, ATF4, GRP78 and VEGF, and reduces NFATc1 in ONFH *in vivo*

**Figure 6:**
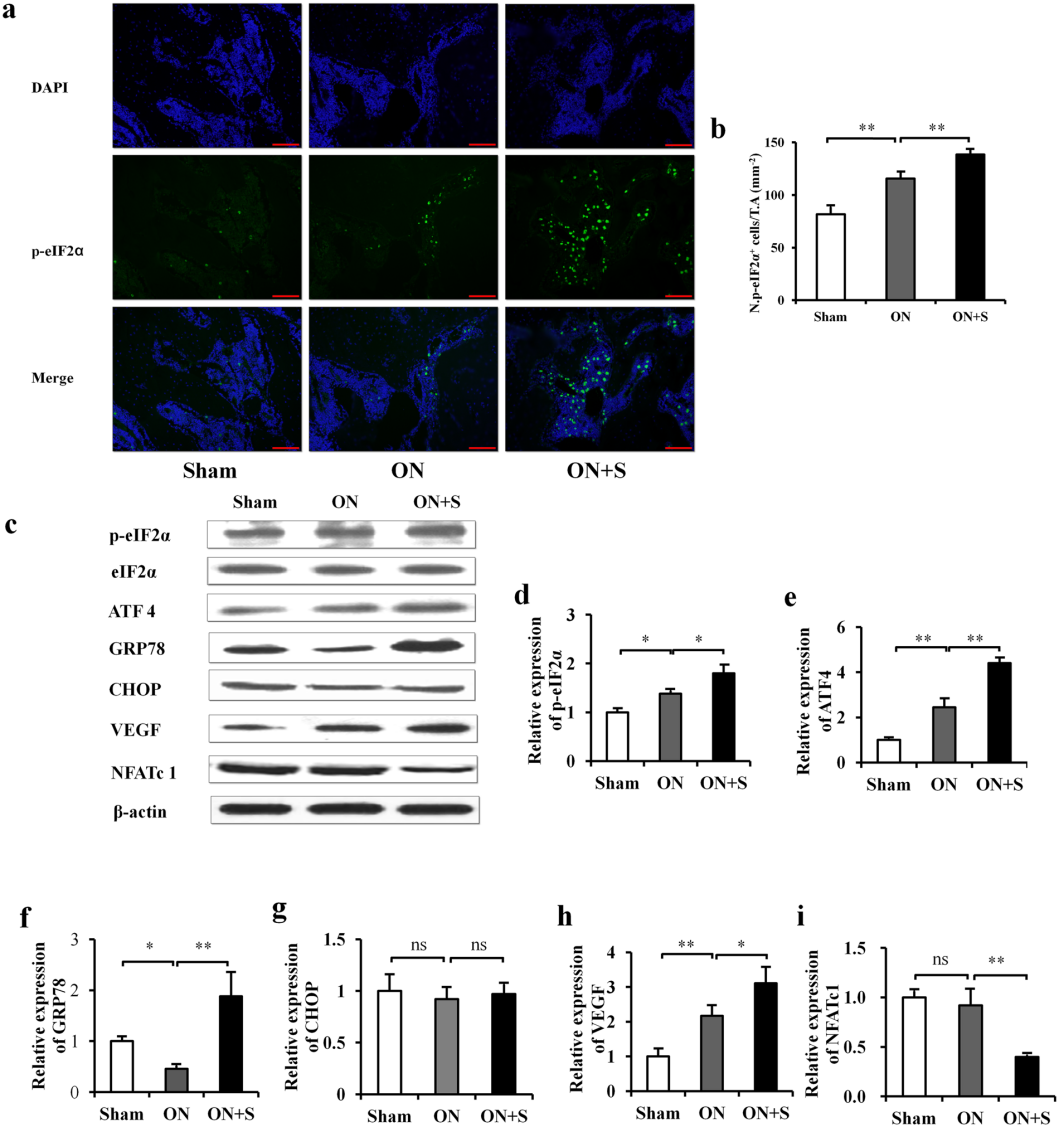
Salubrinal increases p-eIF2α, ATF4, GRP78, CHOP and VEGF, and reduces NFATc1 in ONFH *in vivo*. (**a**) Representative histological immunofluorescence images of femoral head from different groups (blue: DAPI, green: p-eIF2α^+^ cells, 200×, Bar = 100 μm). (**b**) Numbers of p-eIF2α^+^ cells were analyzed. (**c**) Representative images of Western blotting in three different groups *in vivo*. The levels of p-eIF2α (**d**), ATF4 (**e**), GRP78 (**f**), CHOP (**g**), VEGF (**h**), and NFATc1 (**i**) are shown. The asterisks (* and **) represent *P* < 0.05, and *P* < 0.01, respectively, and “ns” indicates *P* > 0.05 (n = 6). (**f**) Proposed mechanism of salubrinal’s action on improving ONFH.

The images of immunofluorescence showed that p-eIF2α positive cells (green) were mainly located in the bone marrow cavity (Fig. 6a). Compared to the sham control group, the ON group increased the expression level of p-eIF2α (*P* < 0.01). The salubrinal-treated group exhibited a further increased level of p-eIF2α (*P* < 0.01; Fig. 6b). Compared to the sham control group, the ON group increased the levels of p-eIF2α, ATF4, and VEGF, and decreased the levels of GRP78. The salubrinal-treated group increased the level of p-eIF2α, ATF4, GRP78 and VEGF, but it presented a lower level of NFATc1 than that the ON group (all *P* < 0.05). The level of CHOP was not significantly different in three groups (all *P* > 0.05) (Fig. 6c–i).

### Correlation exists among p-eIF2α, angiogenesis, vessel remodeling, and bone healing

To evaluate the correlation among parameters in salubrinal’s improving of ONFH symptoms, we selected the p-eIF2α level for representing salubrinal’s effect, VEGF level for angiogenesis, vessel number for vessel remodeling, and osteoblast number and osteoclast surface for bone healing. The result indicated that the level of p-eIF2α was positively correlated with the level of VEGF (r = 0.902, *P* < 0.001) as well as the osteoblast number (r = 0.672, *P* < 0.01). Furthermore, the vessel number was positively correlated with the osteoblast number (r = 0.613, *P* < 0.05) and negatively correlated with the osteoclast surface (r = −0.829, *P* < 0.001).

## Discussion

Interruption of the blood supply to the femoral head is a prime cause of ONFH induction^31^. In the rat model of ONFH, ischemic osteonecrosis was surgically induced and the effect of salubrinal was investigated. Compared to the sham control group, the ON group decreased BV/TV, Tb.N, Tb.Th, B.Ar/T.Ar and femoral BMD/BMC, with an increase in empty lacunae and Tb.Sp. As reported in the previous ONFH studies^12, 16^, the results herein are consistent with significant bone loss in the femoral head. ONFH also presented the decrease in blood perfusion and the level of VEGF^16, 32, 33^. It presented the fewer vessel number and smaller vessel area than those in the sham control group. In orthopaedic diseases such as osteoporosis, osteoarthritis, and osteonecrosis, osteoclastogenesis is abnormally activated^34–38^. The result in this study revealed that osteoclastogenesis was also activated in the femoral head of ONFH. The number of TRAP-positive cells was significantly increased in the bone surface of ONFH. Furthermore, osteonecrosis activated the differentiation, migration, adhesion, and activity of osteoclasts, although osteoblast differentiation in the sham control group was not markedly different from that in the ON group.

ONFH primarily results from ischemia/hypoxia to the femoral head. One of the cellular manifestations of ischemia/hypoxia is the ER stress. The ER stress is known to lead to three major signaling pathways, such as the PERK-ATF4 axis, activating transcription factor 6 (ATF6) axis, and inositol-requiring enzyme-1a/X-box binding protein 1 (IRE1a/XBP1) axis^1, 26^. A unique feature in disease conditions such as ONFH is that the ER stress may act as a two-edged sword. While a moderate level may assist the reestablishment of cellular homeostasis, the prolonged and unmitigated stress may lead to apoptosis^1, 26^. In the present model of ONFH, the ischemic femoral head was unable to acquire an adequate supply of blood, oxygen, or nutrients^20^, and the excessive ER stress was induced. The result in this study showed that osteonecrosis elevated the level of p-eIF2α and ATF4. The elevated level of ATF4, which is induced by the ER stress^1, 26^, is reported to promote osteoclastogenesis^39, 40^. In the ONFH model, we also observed stimulation in osteoclastogenesis in the ischemic femoral head. Correlation analysis indicated that blood perfusion was negatively associated with osteoclastogenesis. Collectively, ischemia in ONFH induced osteoclastogenesis and interrupted bone healing by inducing excessive ER stress.

Salubrinal elevates the level of p-eIF2α and activates eIF2α mediated signaling^25^, and it stimulates bone metabolism in orthopaedic diseases^27–29^. We recently presented several lines of evidence that osteoclastogenesis in osteoporosis was associated with the ER stress, and salubrinal suppressed unloading-induced bone loss^30^. The result in this study showed that salubrinal prevented bone loss in ONFH by suppressing osteoclast activity and promoting osteoblast development. Salubrinal is reported to suppress osteoclastogenesis by reducing the level of NFATc1, and promote osteoblastogenesis by increasing the level of ATF4. The results with NFATc1 and ATF4 in this study are consistent with those in the previous studies^27, 41^.

ATF4 has a dual function in the ER stress pathway. It may serve as a pro-survival factor by promoting transcription of ER chaperones such as GRP78 and reducing the accumulation of misfolded proteins. Alternatively, it may lead to apoptosis by inducing CHOP, which is a pro-apoptotic factor^26^. To evaluate the degree and effect of ER stress in the present ONFH model, we determined the levels of GRP78 and CHOP. Although the level of CHOP was not detectably different, the level of GRP78 in the ONFH model was significantly decreased. The reduction of GRP78 suggests that the ER stress was maintained at the unmitigated level, and the chaperones became unavailable under the prolonged ER stress. Salubrinal attenuated prolonged ER stress through the eIF2α - ATF4 - GRP78 regulatory axis.

Regarding the effects of salubrinal on angiogenesis, we determined the level of VEGF and its action on endothelial cells. The result showed that p-eIF2α increased the expression of ATF4^1, 26^, and ATF4 promoted the secretion of VEGF, a stimulator of angiogenesis^26, 42–46^. Salubrinal protected the proliferation, migration, and angiogenesis of HUVECs against the excessive ER stress, which was induced by tunicamycin. Salubrinal also increased the p-eIF2α level in HUVECs and increased those of p-eIF2α, ATF4, and VEGF *in vivo*.

To further evaluate the effect of salubrinal on the ER stress, we employed Calyculin A (CA) as a specific inhibitor of PP1^25^. Tunicamycin inhibited the cell viability of MC3T3-E1 cells, while salubrinal and CA suppressed tunicamycin’s inhibition. To investigate the in-depth mechanism of salubrinal’s action on the ER stress, RNA interference with eIF2α siRNA was conducted. Salubrinal rescued the cell viability of MC3T3-E1 and HUVECs under the ER stress. Transfection of eIF2α siRNA blocked the effect of salubrinal on cells viability under ER stress. Tunicamycin significantly inhibited the levels of ALP and RUNX2, while salubrinal increased those of p-eIF2α, ATF4, ALP, and RUNX2. Treatment with eIF2α siRNA, however, blocked the effects of salubrinal on the levels of p-eIF2α, ATF4, ALP, and RUNX2, as well as migration and angiogenesis of HUVECs in the presence of tunicamycin. These results indicated that salubrinal protects osteoblasts and endothelial cells against the excessive ER stress by modulating the eIF2α signal pathway.

**Figure 7:**
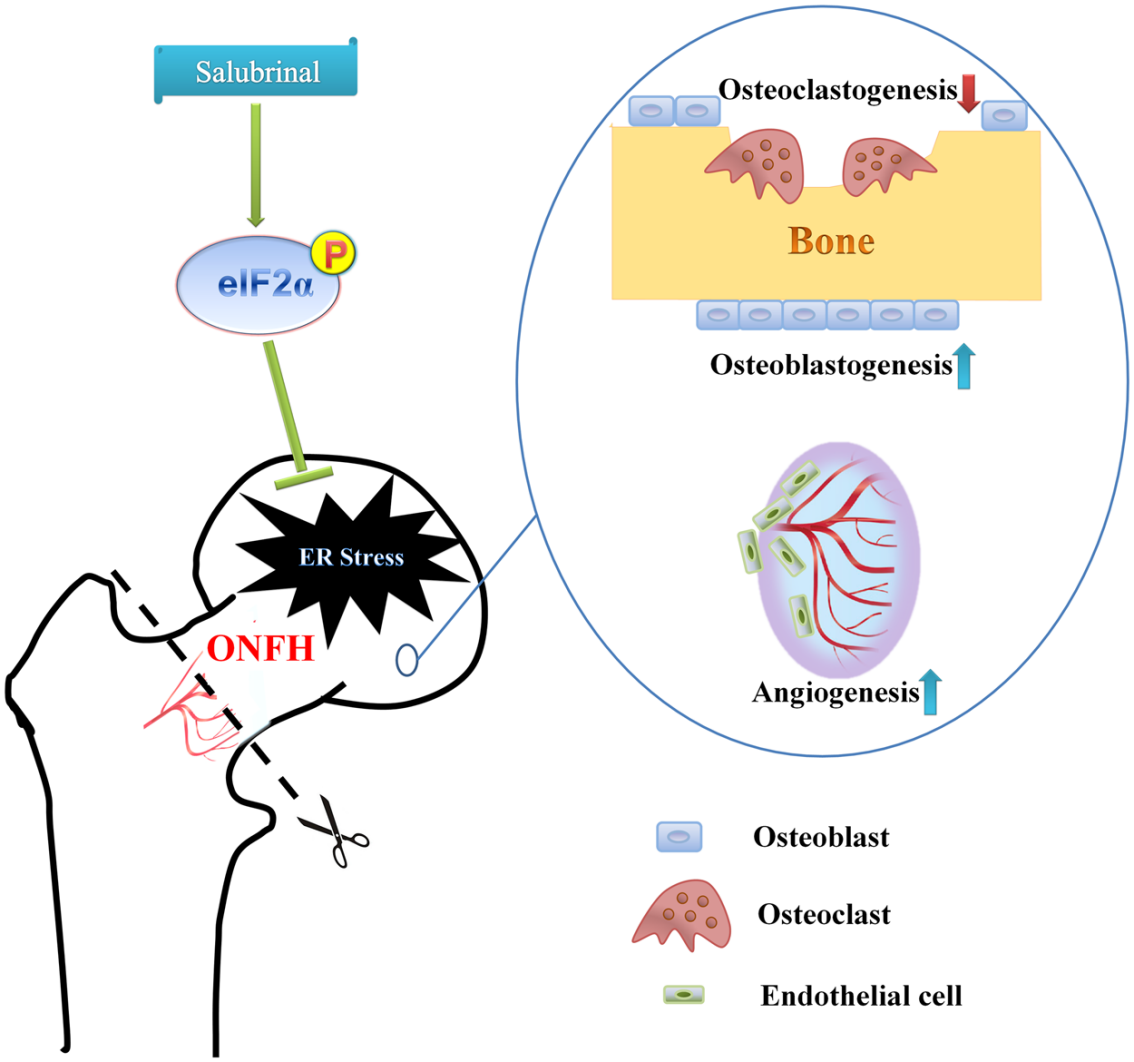
Schematic description of eIF2α signaling regulates ONFH. Ischemia is an inducer of the ER stress in the surgery-induced rat ONFH model. Salubrinal promoted osteoblastogenesis, suppressed osteoclastogenesis, and stimulated angiogenesis by elevating the level of p-eIF2α through suppressing ER stress signaling in ONFH.

We evaluated toxicity of salubrinal and CA in MC3T3-E1 cells. The result indicated that the non-toxic dose of salubrinal and CA were no more than 5 μM and 0.2 nM, respectively. Collectively, salubrinal not only protected osteogenesis against the ER stress but also had lower toxicity than CA. Furthermore, salubrinal promoted osteoblastogenesis, suppressed osteoclastogenesis, and stimulated angiogenesis through suppressing ER stress signaling by elevating the level of p-eIF2α in ONFH (Fig. 7). In conclusion, ischemia is an inducer of the ER stress in the surgery-induced rat ONFH model, and an excessive ER stress induces osteoclastogenesis and inhibits angiogenesis. Alleviating the ER stress is a potential option to improve symptoms associated with ONFH. The present study suggests that the ER stress is an important pathological outcome in the surgery-induced ONFH model, in which salubrinal improves the ONFH symptoms by alleviating the ER stress.

## Materials and Methods

### Preparation of animals and materials

Male Sprague-Dawley rats (~14 weeks of age, Animal Center of Academy of Military Medical Sciences, China) were housed on a 12:12 h light-dark cycle under the pathogen-free conditions, and were fed with free access to food and water. All experiments were carried out according to the National Institutes of Health Guide for Care and Use of Laboratory Animals, and were approved by the Ethics Committee of the Tianjin Medical University. Salubrinal was purchased from Tocris Bioscience (Bristol, UK). Murine macrophage-colony stimulating factor (M-CSF) and murine receptor activator of nuclear factor kappa-B ligand (RANKL) were purchased from PeproTech (Rocky Hills, NC, USA). Human umbilical vein endothelial cells (HUVECs) and endothelial cell growth medium-2 (EGM2) were purchased from Lonza (Walkersville, MD, USA). Lipofectamine 2000 Reagent, Minimum essential medium alpha (MEM-α), Dulbecco’s Modified Eagle’s Medium (DMEM), penicillin, streptomycin, fetal bovine serum (FBS) and trypsin were purchased from Invitrogen (Carlsbad, CA, USA). Scrambled siRNA and eIF2α siRNA were from GenePharma (Shanghai, china). Matrigel was purchased from Life Sciences (Tewksbury, MA, USA). Primary antibodies specific to p-eIF2α, ATF4, RUNX2, CHOP and eIF2α were purchased from Cell Signaling (Danvers, MA, USA). The antibody for GRP78 was from Thermo (Rockford, AL, USA). The antibodies for ALP, nuclear factor of activated T-cells, cytoplasmic 1 (NFATc1), vascular endothelial growth factor (VEGF), and β-actin were purchased from Abcam (Cambridge, MA, USA). Other chemicals were purchased from Sigma (St. Louis, MO, USA).

### Experimental design

Sixty rats were randomly divided into 4 groups: sham control group (Sham, n = 18), osteonecrosis group (ON, n = 18), salubrinal-treated osteonecrosis group (ON + S, n = 18) and salubrinal-treated sham control group (Sham + S, n = 6). Ischemic osteonecrosis of the bilateral femoral heads were induced according to the procedure previously described^12, 47^. Briefly, after anesthesia, a longitudinal incision was made on the skin over the greater trochanter. The gluteus maximus muscle and the gluteus medius muscle were separated from the bone. The joint capsule was transected and the femoral head was dislocated. ONFH was induced by transecting the ligamentum teres and tightly placing a ligature (#3-0 Vicryl, Ethicon) around the femoral neck. After the femoral head was relocated, muscles and skin were sutured with #3-0 and #4-0 stitches, respectively. For sham control and salubrinal-treated sham control groups, rats received the same procedure except for ligating the femoral neck and transecting the joint capsule. In addition, analgesia and antibiotic prophylaxis were applied for the first three postoperative days. After surgery, a dosage of 0.05 mg/kg salubrinal (resolved in propylene glycol) was administered subcutaneously every day for 4 wks to the salubrinal treatment group and salubrinal-treated sham control group^28^. The sham control and osteonecrosis groups were given the vehicle. All animals were euthanised in 4 wks after operation. Bone marrow-derived cells were collected from tibias, and femurs were used for imaging and histology.

### Micro-computed tomography (μ-CT) and peripheral dual-energy X-ray absorptiometry (pDEXA) analysis

Formalin-fixed femurs were examined by μ-CT scanner (VivaCT 40; Scanco Medical AG, Bassersdorf, Switzerland) at a voxel size of 10.5 μm. The voxel of interest (VOI) was comprised of 600 CT slices. We determined fractional bone volume (BV/TV), trabecular number (Tb.N), trabecular thickness (Tb.Th), and trabecular spacing (Tb.Sp). Furthermore, bone mineral density (BMD) and bone mineral content (BMC) of the femur were determined with pDEXA, and changes in BMD and BMC (%) were determined^48^.

### Histological assays

Femoral heads were fixed with 10% neutral buffered formalin for 2 days, and decalcified with 10% ethylenediaminetetraacetic acid (EDTA, pH 7.4) for 40 days. Samples were embedded in paraffin and cut into 5-μm thickness coronal slices as previously described^47^. The ratio of trabecular bone area to tissue area (B.Ar/T.Ar), and the percentage of empty lacunae in bone tissue were analyzed using hematoxylin-eosin (H&E) staining^21, 49^. Tartrate resistant acid phosphatase (TRAP) staining were used to detect the percentage of osteoclasts^49, 50^. MacNeal’s staining were used to determine the number of osteoblasts^49, 51, 52^. For all histological assays, three sections were assessed per femoral head and five 100–400× fields were randomly selected per section. All graphics were measured and analyzed by Cellsense Standard software (Olympus).

### Isolation of bone marrow-derived cells and osteoclast development assay

Bone marrow-derived cells were collected as described previously^47, 53^. After euthanasia, we flushed the bone marrow of tibias with DMEM (containing 2% FBS). The cells were separated with Ficoll low-density gradient centrifugation. We seeded cells onto 96-well plates at a density of 1 × 10^5^ cells/well, and cultured in MEM-α supplemented with 30 ng/ml M-CSF and 20 ng/ml RANKL for 3 days. On day 4, the culture medium was replaced by MEM-α supplemented with 30 ng/ml M-CSF and 60 ng/ml RANKL, and cells were grown for additional 3 days. Osteoclasts were identified using a TRAP staining kit (Sigma-Aldrich). TRAP-positive multinuclear cells (more than 3 nuclei) were identified as matured osteoclasts^54^. In all cell culture experiments, five random fields per well were selected and photographed for analysis.

Colony-forming unit-macrophage/mononuclear (CFU-M) and colony-forming unit-granulocyte-macrophage (CFU-GM) assays were performed. We seeded bone marrow-derived cells onto 6-well plates at a density of 2.5 × 10^4^ cells/well. The culture medium was composed of methylcellulose with 30 ng/ml M-CSF and 20 ng/ml RANKL. Cells were cultured at 37 °C in a 5% CO_2_ incubator for 7 days. The colony numbers in CFU-M/CFU-GM were converted to the numbers per tibia^53, 55^.

Migration of pre-osteoclasts was performed with a transwell device as described previously^53^. To achieve pre-osteoclasts, we seeded bone marrow-derived cells (2 × 10^6^ cells/ml in 6-well plates) in MEM-α supplemented 10% FBS, 30 ng/ml M-CSF and 20 ng/ml RANKL for 4 days. For migration assay, the pre-osteoclasts (1 × 10^5^ cells/well) were resuspended onto the upper chamber of transwells and allowed to migrate to the bottom chamber through an 8-μm polycarbonate filter coated with vitronectin (Takara Bio Inc., Otsu, Shigma, Japan). The medium in the upper chamber was replaced by MEM-α without serum, and MEM-α (consisting of 1% bovine serum albumin and 30 ng/ml M-CSF) were loaded onto the lower chamber. After 6 h incubation, the number of migrated pre-osteoclasts was counted. For adhesion assay, the pre-osteoclasts (1 × 10^5^ cells/well) were seeded onto 96-well plates which were coated with vitronectin in MEM-α supplemented with 30 ng/ml M-CSF. The number of adherent pre-osteoclasts was counted after 30-min incubation^56^. For pit formation assay, a UV-sterilized bovine cortical bone slices (120 μm thick) were used in bottom of the 24-well plate^54^. We seeded bone marrow-derived cells onto the plate (1 × 10^5^ cells/well), and cultured cells with MEM-α supplemented 10% FBS, 30 ng/ml M-CSF and 20 ng/ml RANKL for two days. On the day 3, the concentration of RANKL was increased to 60 ng/ml, and the medium was changed every 2 days. On day 10, the percentages of the pit area to total area in each field view were calculated. In all above experiments, cells were stained with crystal violet, and five random fields per well were selected and photographed for analysis.

### Osteoblast differentiation and colony-forming unit-fibroblast (CFU-F) assays

In an osteoblast differentiation assay, bone marrow-derived cells were cultured onto 6-well plates (2 × 10^6^ cells/well) with osteogenic differentiation medium, and the medium was changed every 2 days. On day 14, cells were stained using an alkaline phosphatase (ALP) staining kit (Sigma) and the percentages of ALP-positive cells were determined^48^.

In a CFU-F assay, bone marrow-derived cells were seeded onto 6-well plates (2 × 10^6^ cells/well) with complete MesenCult medium. The medium was changed every 2 days for 14 days. Then, fibroblasts were stained using a HEMA-3 quick staining kit (Fisher Scientific, Waltham, MA, USA) and the colonies with more than 50 cells were counted^57, 58^.

### Immunofluorescence

Immunofluorescence analysis of the femoral head sections was performed as described previously^50, 59^. Bone sections were permeabilized in 0.3% Triton X-100 for 10 min, and blocked in 10% goat serum at room temperature for 30 min. Furthermore, the sections were incubated with special primary antibodies (p-eIF2α 1:100) overnight at 4 °C. Subsequently, the samples were incubated with secondary antibodies conjugated with fluorescence at room temperature for 1.5 h (avoiding light). The expression of p-eIF2α was determined, in which the nuclei were counterstained with DAPI.

### Blood perfusion assay

To analyze blood perfusion of the femoral head with ONFH, an ink perfusion assay was performed using Chinese ink as previously described^33, 47^. The heart of rat was exposed by surgery after anesthesia. A needle was inserted into the left ventricle for ink infusion, and the right atrium was opened. The circulation were flushed by a heparin-saline solution (25,000 units in 250 ml of 0.9% sodium chloride) until clear liquid flowed from the right atrium. With that, 5% gelatin/ink solution (the ratio between Chinese ink and water was 1:1) was injected into the circulation until the skin of animals became uniformly black. The femoral heads were harvested, and fixed with in 10% neutral buffered formalin for 2 days, and were decalcified in 10% ethylenediaminetetraacetic acid (EDTA, pH 7.4) for 40 days. Samples were embedded in paraffin and cut into 25-μm thickness slices. The quantification of vessel numbers was the vessel numbers in each 200× field view, and vessel area percentages was the ratio of vessel area to total area in each 200× field view.

### Transfection

Transient knockdown of endogenous eIF2α was achieved by siRNA which targeting eIF2α. Transfection was performed with Lipofectamine 2000 Reagent (Invitrogen) following the manufacturer’s protocol. Briefly, cells were seeded in plates the day before transfection to ensure a suitable cell confluent on the day of transfection. Scrambled siRNA 5′-ACGUGACACGUUCGGAGATT-3′ or eIF2α siRNA 5′-AAGCUACUUCAAUAUCU CTT-3′ were used for transfection in antibiotic free Opti-MEM medium (Invitrogen). Two days after transfection, the transfected cells were then ready for the following experiments.

### Cell viability assay

MTT assay was used to evaluate the cell viability as previously described^60^. HUVECs were seeded in 96-well plates at a density of 1 × 10^4^ cells/well with EGM2. Four hours later, cells were transfected with 20 nM eIF2α siRNA or scrambled siRNA, and treated with various treatment factors or vehicle, respectively. After 48 h, the absorbance at 570 nm was detected using a μQuant universal microplate spectrophotometer (Bio-tek, Winooski, USA).

### Migration and wound-healing assays of HUVECs

Migration of HUVECs was evaluated with trans-well equipment as described previously^50^. HUVECs were transfected with 20 nM eIF2α siRNA or scrambled siRNA. 48 h later, cells (2 × 10^4^ cells/well) were suspended in serum-free medium in the upper chambers and incubated them with various treatment factors or vehicle. EGM2 (consisting of 20% FBS) were loaded onto the lower chamber. The number of cells that migrated to the lower surface in 12 h was counted using crystal violet staining. In a wound-healing assay, HUVECs were transfected with 20 nM eIF2α siRNA or scrambled siRNA in 6-well plates for 48 h until grown to 100% confluence. Using a 200-μl pipette tip, a scratch was made on a cell monolayer. The width of scratch was measured on 0 h and 20 h, respectively, and the percentages of scratch recovery were calculated^60^.

### Tube formation assay of HUVECs

HUVECs were transfected with 20 nM eIF2α siRNA or scrambled siRNA for 48 h. We coated 24-well plates with Matrigel and incubated them to polymerize at 37 °C for 45 min. HUVECs (2 × 10^5^ cells/well) were then seeded on the polymerized Matrigel. Cells were incubated with EGM2-containing treatment agents or vehicle for 12 h to stimulate tube formation^50^. Images of tubes were taken, and the cumulative tube lengths were calculated.

### Osteogenesis assay *in vitro*

Cell viability (MTT assay) and the expression levels of osteoblast markers were determined under the tunicamycin-induced ER stress. MC3T3-E1 cells were seeded in 96-well plates at a density of 1 × 10^4^ cells/well in DMEM with 10% FBS. Four hours later, cells were transfected and were exposed to the treatment agents or vehicle. After 48 h, the absorbance at 570 nm was detected in the MTT assay. In addition, 48 h after transfection and administration, the expression levels of p-eIF2α, ATF4, ALP, and RUNX2 inMC3T3-E1 cells were determined by Western blot.

### Western blot assay

Bone tissue powders from the femurs or cells were lysed in a RIPA lysis buffer, which contained the inhibitors of proteases and phosphatases (Roche Diagnostics GmbH, Mannheim, Germany). The levels of p-eIF2α, eIF2α, ALP, RUNX2, ATF4, NFATc1, VEGF, and β-actin were determined by Western blot as described previously^61^. The samples were resolved in sodium dodecyl sulfate-polyacrylamide gel and transferred onto the polyvinylidene difluoride membranes. The membranes with primary antibodies were incubated overnight at 4 °C (p-eIF2α 1:2000, eIF2α 1:4000, ALP 1:2000, RUNX2 1:1000, ATF4 1:1000, NFATc1 1:2000, VEGF 1:2000, GRP78 1:20000, CHOP 1:1000 and β-actin 1:10000). Soon afterwards, the membranes were washed and incubated with horseradish peroxidase-conjugated secondary antibody (1:20000). Enhanced chemiluminescence was used to assess protein expression. Image acquisition and analysis software (Bio-rad) were used to quantify band intensities.

### Statistical analysis

Data were expressed as mean ± standard deviation (SD). Data were analyzed with independent-sample *t* test (for two groups) or one-way ANOVA (for more than two groups). Correlation analysis among parameters was performed by Pearson correlation coefficient test. Statistical significance was assumed at *P* < 0.05. The asterisks (*, **, and ***) represent *P* < 0.05, *P* < 0.01, and *P* < 0.001, respectively.

## Electronic supplementary material

Supplementary Information
