# Supplementary material for: eIF2α signaling regulates ischemic osteonecrosis through endoplasmic reticulum stress
Source: Sci Rep. 2017 Jul 11;7:5062. doi: 10.1038/s41598-017-05488-6 (PMC5505953; doi:10.1038/s41598-017-05488-6)
Supplement: Supplementary file 1 — Supplementary Information [file 41598_2017_5488_MOESM1_ESM.doc]

**Supplementary Information**

**eIF2α** **signaling regulates ischemic osteonecrosis through endoplasmic reticulum stress**

Daquan Liu, Yunlong Zhang, Xinle Li, Jie Li, Shuang Yang, Xiaoxue Xing, Guanwei Fan, Hiroki Yokota & Ping Zhang

**Supplemental Fig. 1.**

**
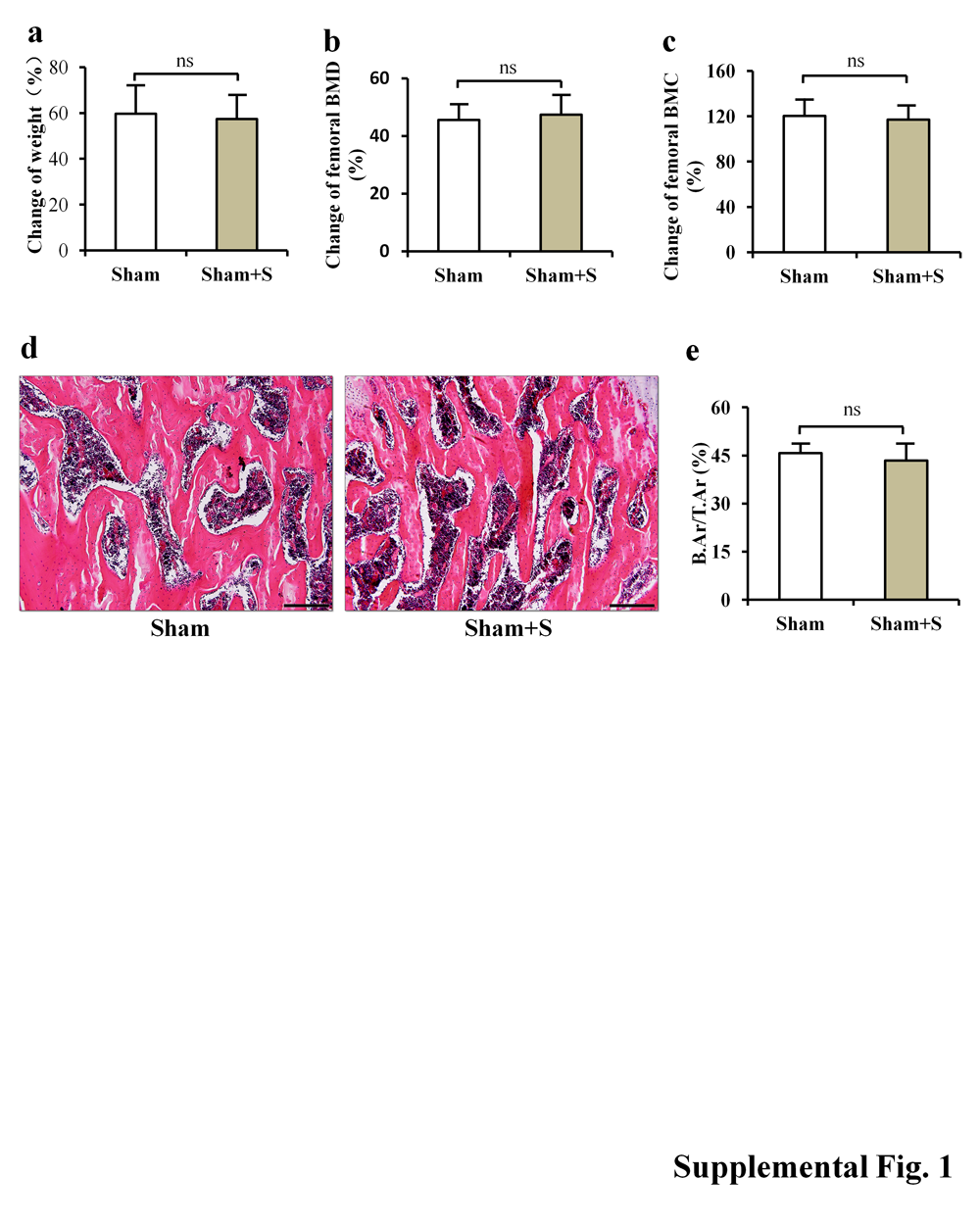
**

**Supplemental Fig. 1. The effects of salubrinal on bone metabolism of normal animals.** (a) Body weight, (b) bone mineral density (BMD) and (c) bone mineral content (BMC) of animals between sham control and salubrinal-treated sham control groups were analyzed. (d) Representative histological images of the femoral head by H&E staining (100×, Bar=200 μm). (e) Ratio of the trabecular bone area to the tissue area (B.Ar/T.Ar). (Sham: sham control group, and Sham+S: salubrinal-treated sham control group).

**Supplemental Fig. 2.**

**
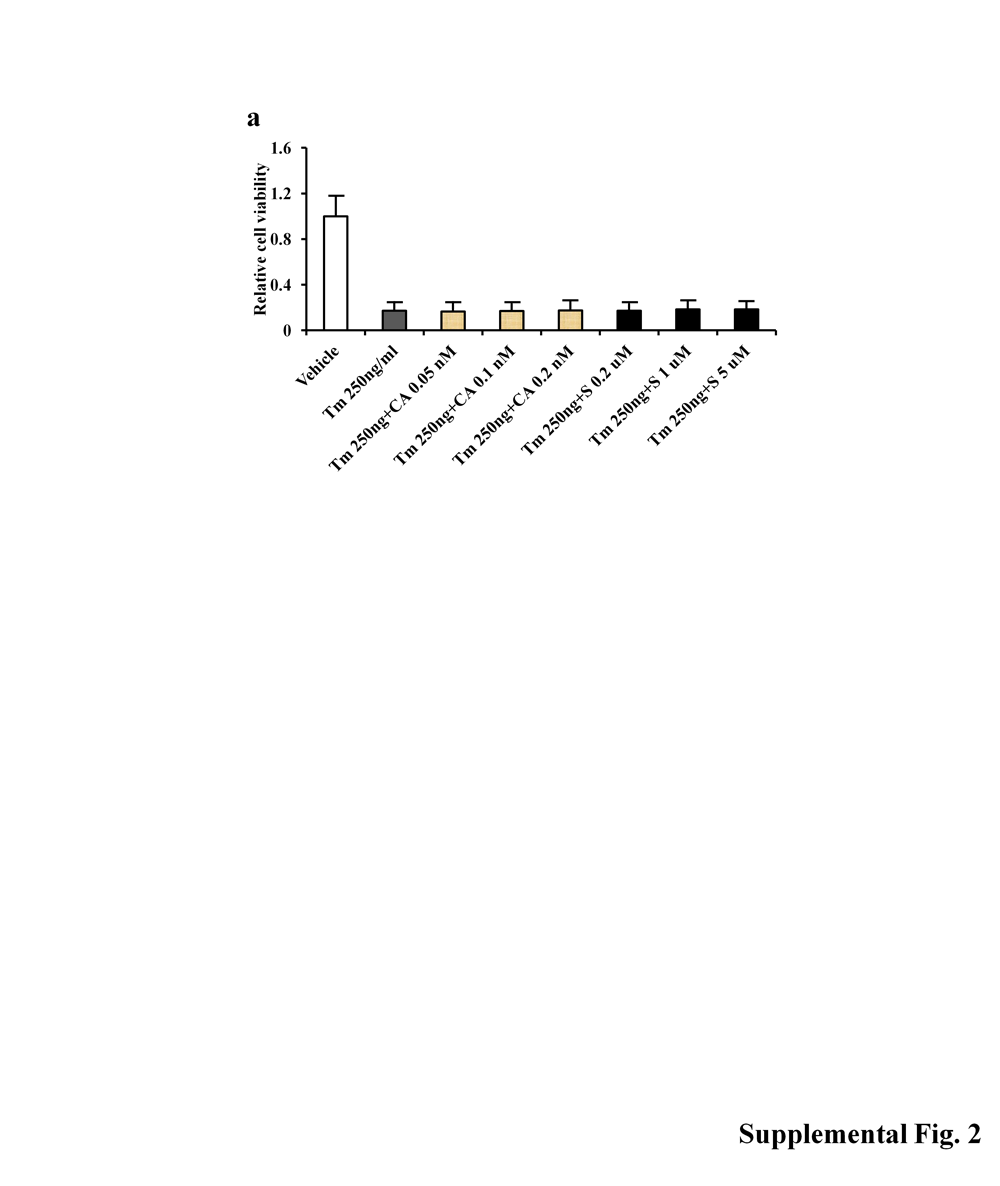
**

**Supplemental Fig. 2.** V**iability of osteoblasts under excessive ER stress condition.** (a) MTT assay for cell viability of MC3T3-E1 cells. The histogram showed relative cell viability from three independent experiments. Salubrinal and CA didn’t rescue the viability of osteoblasts which are treated by higher concentrations tunicamycin (Tm: Tunicamycin, S: Salubrinal, and CA: Calyculin A).

**Supplemental Fig. 3.**


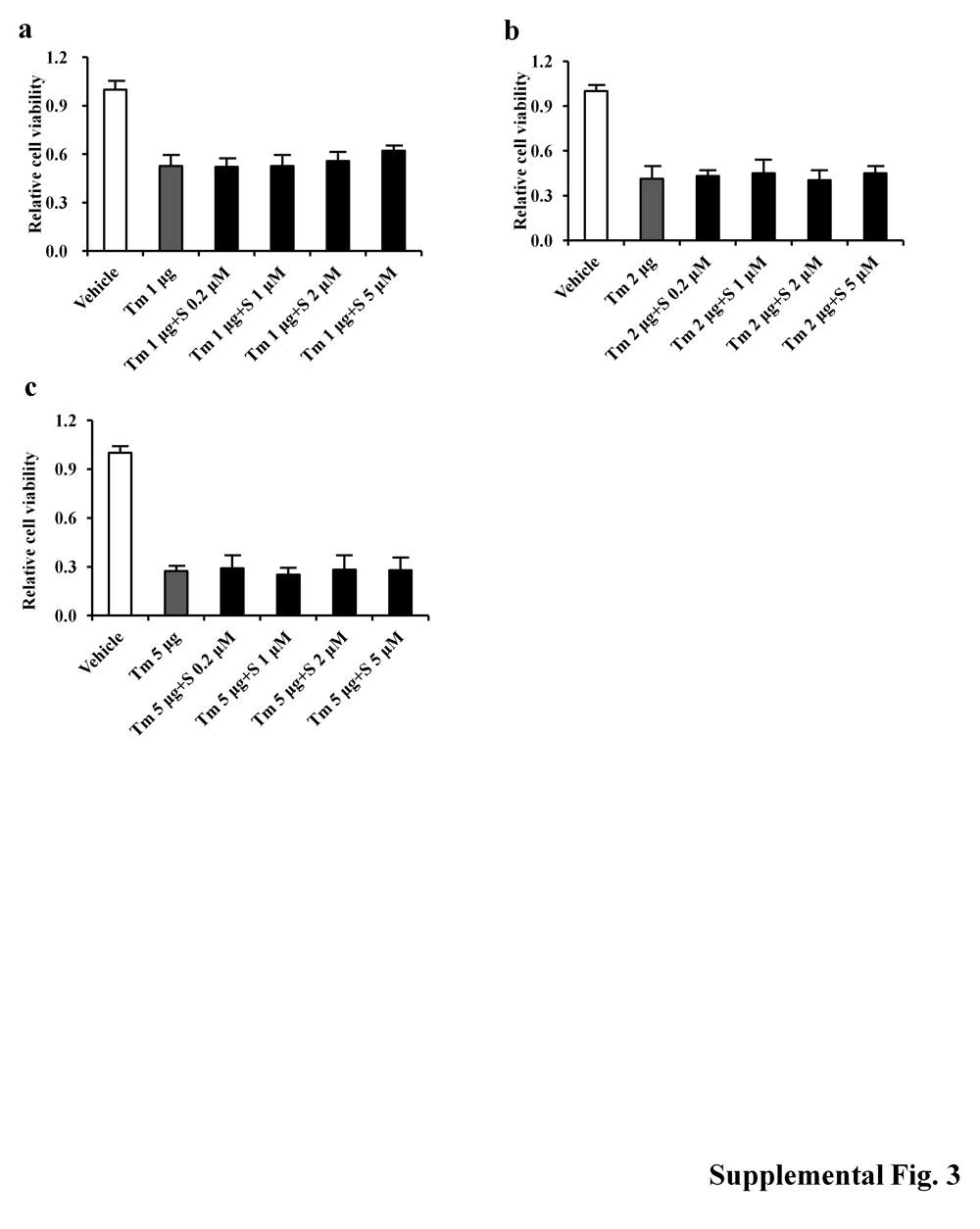


**Supplemental Fig. 3. Viability of endothelial cells under excessive ER stress condition.** (a-c) MTT assay for cell viability of HUVECs. The histogram showed relative cell viability from three independent experiments. Salubrinal does not rescue the viability of endothelial cells which are treated by higher concentrations tunicamycin. (S: Salubrinal, and Tm: Tunicamycin).
